# Clinical and functional characterization of a long survivor congenital titinopathy patient with a novel metatranscript-only titin variant

**DOI:** 10.1186/s40478-023-01539-4

**Published:** 2023-03-21

**Authors:** Nastasia Cardone, Melissa Moula, Rianne J. Baelde, Ariane Biquand, Marcello Villanova, Corinne Metay, Chiara Fiorillo, Serena Baratto, Luciano Merlini, Patrizia Sabatelli, Norma B. Romero, Frederic Relaix, François Jérôme Authier, Valentina Taglietti, Marco Savarese, Josine de Winter, Coen Ottenheijm, Isabelle Richard, Edoardo Malfatti

**Affiliations:** 1 Univ Paris-Est Créteil, INSERM, U955 IMRB, F-94010 Créteil, France; 2 Amsterdam UMC location Vrije Universiteit Amsterdam, Physiology, De Boelelaan 1117, Amsterdam, Netherlands; 3 Genethon, 91000 Evry, France; 4 Neuromuscular Unit, Presidio Ospedaliero Accreditato Villa Bellombra, Bologna, Italy; 5 Unité Fonctionnelle de Cardiogénétique et Myogénétique moléculaire et cellulaire. Centre de Génétique Moléculaire et Chromosomique et INSERM UMRS 974, Institut de Myologie. Groupe Hospitalier La Pitié-Salpêtrière-Charles Foix, Paris, INSERM UMRS1166, Sorbonne Université, Paris, France; 6 Neurologia Pediatrica e Malattie Muscolari, Istituto G.Gaslini, Genoa, Italy; 7 Department of Biomedical and Neuromotor Sciences, University of Bologna, 40126 Bologna, Italy; 8 CNR, Institute of Molecular Genetics “Luigi Luca Cavalli Sforza” -Unit of Bologna, Bologna, Italy; 9 IRCCS-Istituto Ortopedico Rizzoli, Bologna, Italy; 10 Neuromuscular Morphology Unit, Myology Institute, GHU Pitié-Salpêtrière, Paris, France; 11 APHP, Centre de Référence de Pathologie Neuromusculaire Nord-Est-Ile-de-France, Henri Mondor Hospital, Créteil, France; 12 Folkhälsan Research Center, Helsinki, Finland

**Keywords:** Congenital myopathy, Titin, Titinopathy, Rigid spine, Metatranscript, N2A titin isoform, Splicing, Single fiber studies

## Abstract

Congenital titinopathies are an emerging group of a potentially severe form of congenital myopathies caused by biallelic mutations in *titin,* encoding the largest existing human protein involved in the formation and stability of sarcomeres. In this study we describe a patient with a congenital myopathy characterized by multiple contractures, a rigid spine, non progressive muscular weakness, and a novel homozygous *TTN* pathogenic variant in a metatranscript-only exon: the c.36400A > T, p.Lys12134*. Muscle biopsies showed increased internalized nuclei, variability in fiber size, mild fibrosis, type 1 fiber predominance, and a slight increase in the number of satellite cells. RNA studies revealed the retention of intron 170 and 171 in the open reading frame, and immunoflourescence and western blot studies, a normal titin content. Single fiber functional studies showed a slight decrease in absolute maximal force and a cross-sectional area with no decreases in tension, suggesting that weakness is not sarcomere-based but due to hypotrophy. Passive properties of single fibers were not affected, but the observed increased calcium sensitivity of force generation might contribute to the contractural phenotype and rigid spine of the patient. Our findings provide evidence for a pathogenic, causative role of a metatranscript-only titin variant in a long survivor congenital titinopathy patient with distal arthrogryposis and rigid spine.

**Supplementary Information:**

The online version contains supplementary material available at 10.1186/s40478-023-01539-4.

## Introduction

**Fig. 1:**
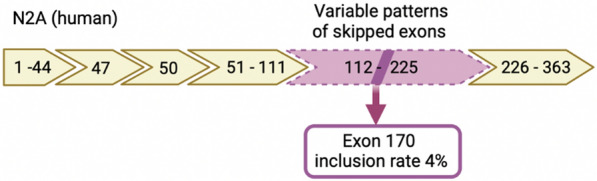
Schematic representation of the N2A human isoform of titin. Yellow boxes represent the normally spliced exons, the white insertions represent the skipped exons. The pink box, represents all the exons potentially expressed in the PEVK region of the isoform, also known as metatranscript-only exons. The inclusion rate of exon 170 in the N2A human isoforms is 4% [6]

Titin is the largest human protein (4200 kDa) involved in the formation and stability of the sarcomeres [1].This giant protein, stretching from the Z-disc to the M-band of the sarcomere, forms the myofilament backbone for the contractile machinery, giving the muscle elastic properties [2]. Titin is encoded by *TTN* gene, one of the largest human genes, spanning 363 exons [1]. Extensive and complex differential splicing of the titin transcript leads the production of several isoforms [3, 4]. The canonical isoform N2A includes 312 exons expressed in skeletal muscle, whilst there are 5 isoforms expressed in cardiac muscle; the largest is N2BA spanning 311 exons [1, 5]. The inferred complete isoform (IC), also referred to as “metatranscript” (NM_001267550), is a theoretical isoform including 363 coding exons, and is the recommended transcript for variant reporting. The exons not included in the canonical isoforms are defined as metatranscript only exons (Fig. 1) [3, 6]. These exons are largely expressed only during embryonic development, and are of variable expression in the post natal setting, which is yet poorly understood [6]. To resume, the metatranscript is a model transcript including all possible in-frame exons for which there is scientific evidence, that are not included in the postnatal isoforms. The inclusion rate of exon 170 in the N2A human isoforms is 4% (Fig. 1) [6]. Mutations in *TTN* gene leads to skeletal and cardiac congenital myopathies, called titinopathies [7]. Congenital titinopathies are an emerging group of a potentially severe form of congenital myopathy caused by biallelic mutations in *titin (TTN)* [1, 2]. Moreover, variants found in exons only included in the inferred complete isoform (IC) were described as pathogenic and referred as ‘metatranscript-only variants’ [6, 8–11]. Here we present the oldest documented patient with a congenital myopathy with rigid spine linked to a pathogenic novel homozygous variant c.36400A > T, p.Lys12134* in exon 170 of *titin*. Deep characterization and thorough functional studies showed a normal titin content and a conserved regenerative muscle capacity whilst an increased calcium sensitivity of force generation could be responsible for the highly contractural phenotype of our patient.

## Methods

The study of the family was approved by the ethical guidelines issued by our institutions for clinical studies in compliance with the Helsinki Declaration. Patient’s parents and patient (P1) gave informed consent for the genetic analysis according to French legislation (Comité de Protection des Personnes Est IV DC-2012-1693). Genomic DNA was extracted from blood by standard methods.

### Targeted gene enrichment, next generation sequencing NGS (high‐throughput sequencing)

P1 and parents DNAs were extracted from peripheral blood with QIAsymphony (Qiagen, Hilden, Germany) and qualitatively checked using Tape Station DNA genomic array (Agilent, Santa Clara, California). Custom targeted gene enrichment and DNA library preparation were performed using the Nimblegen EZ choice probes and Kappa HTP Library preparation kit according to the manufacturer’s instructions (Nimblegen, Roche Diagnostics, Madison, Wisconsin). A specific custom panel of 30 genes was designed including genes associated with retractile myopathies. The RefSeq coding sequences were determined as consensual for genetic diagnosis within a French nationwide working group [12]. The targeted regions include all coding exons and ± 50 base pairs of flanking intronic regions of 30 genes known to be involved in retractile myopathies (Additional file 1: Table S1). Paired-end sequencing was performed on a 250 cycle Flow Cell (Illumina, Santa Cruz, California) using the Illumina MiSeq platform. Eight librairies were multiplexed per run.

### Variants interpretation

Pathogenicity of variant was determined according to current ACMG guidelines that recommend classifying variants into five categories: (a) pathogenic, (b) likely pathogenic, (c) uncertain significance, (d) likely benign or (d) benign. The variant was filtered out according to their allele frequency (≤ 1%) as reported in the GnomAD database (http://gnomad.broadinstitute.org/). We then evaluated the variant considering a careful review of the literature, the location of the variant in the gene and the resulting corresponding protein, the in silico prediction tools (Polyphen2, SIFT, GVGD and CADD for missense variants (http://fraternalilab.kcl.ac.uk/TITINdb/position/IC for missense *TTN* variants) and SpliceSiteFinder like, MaxEntScan, NNSPLICE, GeneSplicer and Human Splicing Finder for splicing variants). In addition, we looked at a local database of pathogenic variants related to our experience on the molecular diagnosis of myopathies.

The pathogenic variant was confirmed by Sanger sequencing in both P1 and parents DNAs.

### Morphological studies

P1 underwent a *vastus lateralis* muscle biopsy at 33 years after informed consent.

For conventional histochemical techniques 10 µm thick cryostat sections were stained with haematoxylin and eosin (H&E), modified Gömöri trichrome (mGT), Periodic acid Schiff technique (PAS), Oil red O, reduced nicotinamide adenine dinucleotide dehydrogenase-tetrazolium reductase (NADH-TR), succinic dehydrogenase (SDH), cytochrome c oxidase (COX), and adenosine triphosphatase (ATPase) preincubated at pH 9.4, 4.63, 4.35. Digital photographs of each biopsy were obtained with a Zeiss AxioCam HRc linked to a Zeiss Axioplan Bright Field Microscope and processed with the Axio Vision 4.4 software (Zeiss, Germany). For immunofluorescence studies the muscles were 7 µm thick cryostat sections were collected on Super Frost Plus slides (Thermo scientific, 10,149,870), permeabilized with Triton 0.5% and blocked in 10% BSA for 30’ at RT. Blocking was followed by an overnight incubation with primary antibodies at 4 °C. The next day, after repetitive washes, slides were incubated with Alexa fluor secondary antibodies for 45 min at 37 °C. For the analysis of fiber type the following primaries were used: MyCH2a (DSHB IIaSc71 IgG1) and MyCH2b (DSHB IIBFF3 IgM). In order to assess the number of satellite cells we used Pax7 (Santa Cruz Biotechnology, sc-81648), ki67 (Abcam, sp6 ab16667). Laminin staining was performed after the secondary antibody incubation, for 1 h at 37 °C using a conjugated antibody (NB300-144AF647). For titin immunofluorescence we used the PEVK 9D10 antibody incubated for 2 h at 37 °C. Histologically normal muscles were used as controls.

### RNA and protein studies

RNA extraction was performed with the Trizol™ method (Thermo Fisher Scientific, Waltham, MA) from frozen tissues. Total RNA was dissolved in 20 µl of RNase-free water and treated with Free DNA kit (Ambion) to remove residual DNA. The quantification was done using a Nanodrop spectrophotometer (ND8000 Labtech, Wilmington Delaware). RNA (1 μg) was reverse-transcribed using the RevertAid H Minus First Strand cDNA Synthesis Kit (Thermo Fisher Scientific) and a mixture of random oligonucleotides and oligo-dT. Real-time PCR was performed using LightCycler480 (Roche, Basel, Switzerland) with 0.2 mM of each primer and 0.1 mM of the probe according to the protocol Absolute QPCR Rox Mix (Thermo Fisher Scientific, Waltham, MA, USA). Two primer pairs used for P1 *TTN* amplification were used. The primer were spanning from exon 170 to exon 172: (Fw: TCGGTGGTGCCTCCTAAA Rv: CAGGAACTACTTCTTTGGGAGG) and from exon 170 to 174 (Fw: AAAGAAAGTGTCGGTGGTG Rv:GGCAACTTCTTTTCTGGGAC). The amplificons were run on a 2% agarose gel. Each experiment was performed in duplicate.

For page electrophoresis, proteins were extracted from frozen tissues grounded in liquid nitrogen by solubilization in urea buffer pH6.8 (8 M urea, 2 M thiourea, 0.05 Tris–HCl, 0.075MDTT, 3% SDS and 0.03% Bromophenol Blue) and 50% glycerol with protease inhibitors (0.04ME64, 0.16MLeupeptin and 0.2MPMSF) at 60 °C for 10 min. Samples were centrifuged at 14,000 g for 5 min and stored at  − 80 °C.

The titin isoform visualization was performed by loading solubilized samples on agarose gels (1%). After electrophoresis at 15 mA per gel for 3 h20, the gels were stained using Coomassie Brilliant Blue and scanned using a commercial scanner. For titin western blotting, solubilized samples were run on a 0.8% agarose gel, then transferred onto PVDF membranes using a semi-dry transfer unit (Trans-Blot Cell, Bio-Rad). Blots were stained with Ponceau S to visualize the total protein transferred. Blots were then probed with the primary antibodies (Z1Z2 *TTN*-1Myomedix; N2A T5650 USbiological; A168-170 #11–96 Myomedix; PEVK 9D10 DSHB) followed by secondary antibodies conjugated with infrared fluorescent dyes.

### Single fiber muscle force measurement

To investigate whether sarcomere dysfunction contributed to the muscle weakness, rigid spine and the contractural phenotype experienced by the patient, contractile measurements on permeabilized muscle fibers were performed. Single muscle fibers were isolated from the *vastus lateralis* biopsy of P1 and histologically normal, age-matched vastus lateralis muscle biopsies, and permeabilized using 10% Triton-X. The permeabilized muscle fibers were mounted between a force transducer and length motor (Permeabilized Fiber System 1400A, Aurora Scientific Inc., Canada) and activated by exogenous calcium solutions. Absolute maximal force, cross sectional area (CSA), maximal tension (maximal force/CSA), calcium sensitivity of force generation and passive stiffness were measured. Single muscle fibers were step wisely stretched (steps of 5% of fiber length determined at sarcomere length 2.5 µm) to measure passive stiffness at incremental sarcomere lengths.

### Data and statistical analysis

Data are expressed as mean ± s.e.m. Values of *p* < 0.05 were considered statistically significant. Prism package (GraphPad Software) were used for data analysis.

### Case presentation

**Fig. 2:**
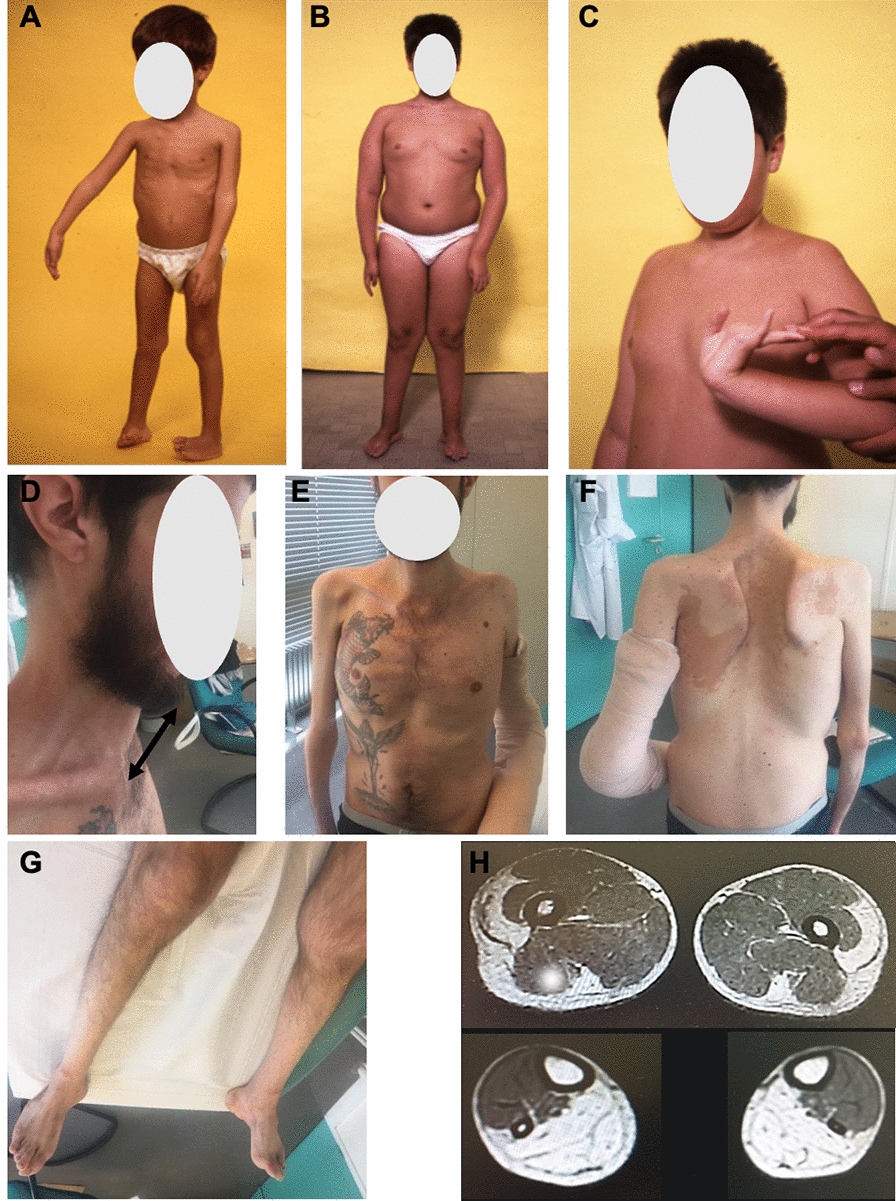
Clinical and imaging features of P1 at 4, 8 and 33 years old. **A** P1 at 4 years. Note the thin muscle bulk, the proximo-distal weakness and atrophy. **B** P1 at 8 years showing elbow contractures, and leg atrophies. **C** Hyperlaxity of wrist joint. **D** Cervical rigid spine. **E** Thoracic deformation and amyotrophy. **F** Scapular winging associated with scoliosis. **G** Prominent distal atrophy. **H** Muscle MRI at thigh and leg level showing bilateral and symmetric patchy adipose replacement of vastus lateralis and semitendinosus muscles, and complete replacement of posterior leg compartment

Patient 1 (P1) is a 36-year-old man second born to non-consanguineous Italian healthy parents coming from the same village of the Gallura region in Sardinia. His elder brother presents a similar clinical phenotype but did not accept to undergo further clinical and genetic investigations. P1 was born at term by vaginal delivery necessitating the utilization of forceps. He was hypotonic at birth, and had distal arthrogryposis with hyperextended wrists, fingers bent toward the ulnar extremity, and varus supinated feet. He showed failure to thrive and moderate motor delay. After delayed gait acquisition, he showed frequent falls, and was never able to run. Examination at 4 years showed a thin muscle bulk, with a flat thoracic cage, and distal weakness and atrophy (Fig. 2A).

From the age of 6 years, he reported the development of progressive joint contractures in elbows and Achilles tendons (Fig. 2B), and he developed spinal rigidity associated with joint hyperlaxity (Fig. 2C). Clinical examination performed in another center showed atrophy and weakness of scapular girdle muscles, and prominent distal atrophy of arm, hands, and legs. Serum Creatine Kinases were normal. Cardiac workup including EKG and ultrasound was normal. The skeletal muscle biopsy of the *tibialis anterior* muscle at 8 years perfomed in another center revealed the presence of multiple internalized nuclei, minicores lesions, and slight type 1 fiber predominance (not shown).

His strength remained stable during adolescence and early adulthood, leading to interruption of follow up and successive referral to our clinic at 33 years. At this time his major complaint was the presence of back pain and reduced joints mobility. Clinical examination, revealed low weight of 37 kg and a BMI of 13.1 kg/m^2^. He presented dysmorphic features with elongated face and high-arched palate without oculo-bulbar involvement, nor facial weakness. There was prominent global amyotrophy, spinal rigidity (Fig. 2D), flat thorax with evident ribs (Fig. 2E), *scapula alata* (Fig. 2F)*,* dorsal right convex scoliosis and lumbar rotation toward the left, and significant, as well as multiple contractures of elbow flexors, hip flexors, wrist and finger flexors. Prominent leg atrophy, bilateral *pes planus* and *valgus* (Fig. 2G) and left hypoplasia of the fourth toe were also observed. Manual muscle testing revealed axial weakness of neck flexor (MRC 3) and mild proximal upper and lower girdle weakness quoting 4 at MRC. Distal weakness of *interossei* hand muscles (MRC 3), and profound finger flexors (MRC 1/5) were also evident. Serum CK level was normal. Muscle MRI of lower limbs performed in another center and reviewed by us, showed symmetrical and bilteral patchy fibro-fatty substitution of *vastus lateralis*, *semitendinosus* and distal part of *vastus intermedius* muscle, as well as complete fibro-fatty substitution of *soleus* and *gastrocnemii* muscles (Fig. 2H). Forced vital capacity was 61% of the predicted values. Nocturnal oximetry was normal. Cardiac workup including EKG and ultrasound did not reveal any alteration.

### Genetic studies

Sequencing of P1 DNA revealed a novel homozygous variant c.36400A > T, p.Lys12134* in exon 170 of *titin*; (NM_001267550)1), corresponding to the PEVK domain. This variant is a stop mutation predicted to create a cryptic donor site in the exon 170 leading to the retention of introns 170 and 171. It was absent in gnomAD, LOVD, Clinvar, PubMed, HGMD Pro, and was considered class 4 (likely pathogenic, PVS1 very strong and PM2 moderate) according to ACMG classification (Varsome search on 10^th^ November 2022). In silico studies using Mobidetails (MPA) software, accessible at https://mobidetails.iurc.montp.inserm.fr/ predicted a pathogenicity MPA score of 10/10 for different criteria as listed: PVS1, a null variant in a gene where LOF is a known mechanism of disease; PM2, absent from controls in the Exome Sequencing Project, 1000 Genomes Project, and Exome Agregation Consortium; PP3, where multiple lines of computational evidence support a deleterious effect on the gene or gene product i.e.conservation, or splicing. The same goes for splice AI and SPiP that predicted an effect on the splicing. Sanger sequencing of exon 170 of *titin* of the parents DNA showed the presence of c.36400A > T, p.Lys12134* in exon 170 of *titin* at hererozygous state.

NGS panel also revealed the the c.62030 T > C, p.(Ile20677Thr) missense variant in *titin* with a 12/248188 allelic frequency in GnomAD, considered highly destabilizing using the https://fraternalilab.kcl.ac.uk/, but presenting moderate scores of bioinformatics prediction tools, not reported in LOVD, and described as VUS in Clinvar. No other pathogenic variants were detected by NGS in the panel included genes except a deletion of 32 to 44 exons in the TNXB gene corresponding to the C-terminal region subjected to structural modifications that is not in keeping with the clinical, histopathologic and imaging phenotype of P1 (Additional file 1: Table S1).

### Morphological studies

**Fig. 3:**
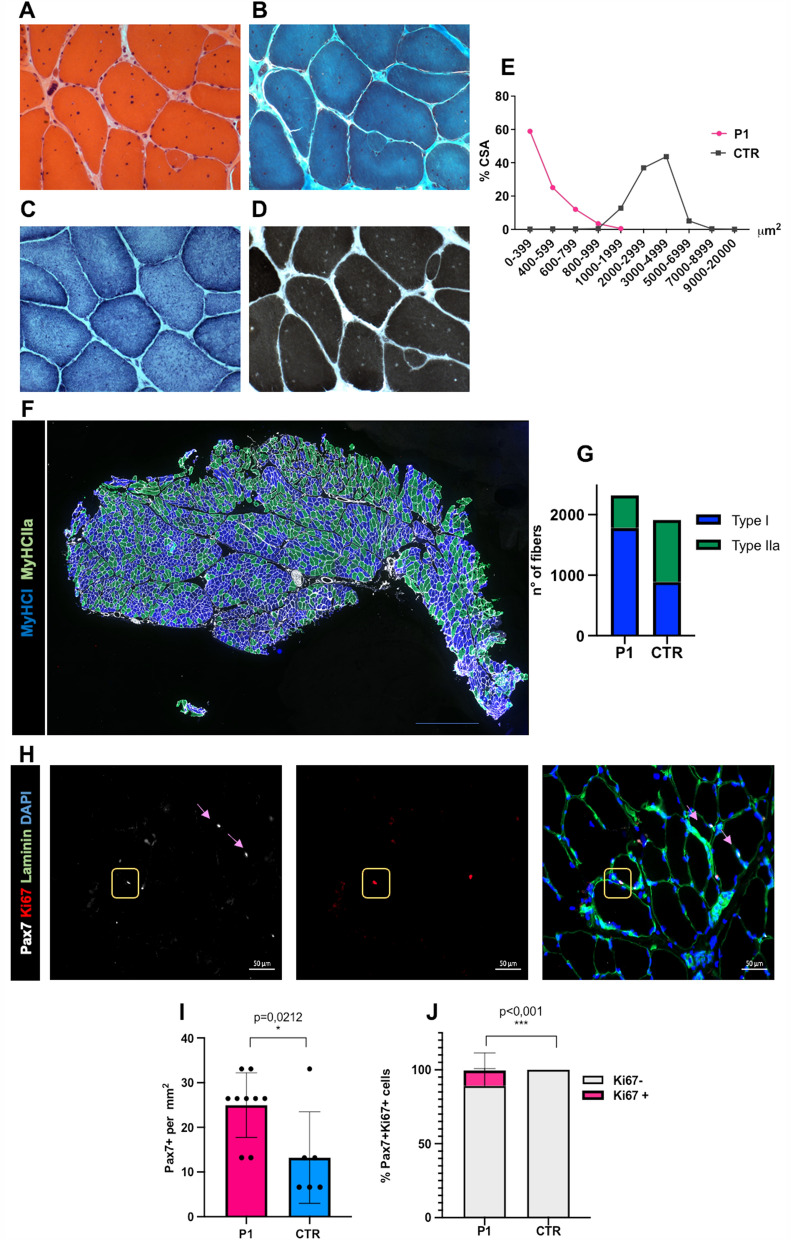
Histopathological findings of the 33 years old vastus laterlais muscle biopsy. **A** Hematoxylin & Eosin, showing fiber size variability, fibers with multiple internalized nuclei and a slight accumulation of endomysial fibrotic tissue. **B** Gömöri trichrome staining showing increased endomysial tissue and multiple internalizations. **C** NADH-TR showing homogenous intermyofibrillary network. **D** ATPase 9.4 showing type 1 fibers predominance. **E** Distribution of fiber size of P1 showing global fibres hypotrophy compared to a control. **F** MyHC I and IIA immunofluorescence. **G** Histogram representing the total number of type 1 and type 2a fibers compared to a control (76.73%). **H** Pax7, Ki67, Laminin, Dapi immunofluorescence. Pax7 positive cells are indicated by pink arrows. Pax7/Ki67 positive cell, indicated by yellow square. **I** Histogram presenting the total number of Pax7/mm^2^ positive cells, showing a slight increase in the number of SCs compared to control. **J** Histogram showing the percentage of Pax7/Ki67 double positive cells on the total of Pax7 positive cells

In order to validate the pathogenic role of the exon 170, c.36400A > T, p.Lys12134* *titin* variant, P1 consented to perform a muscle biopsy in the *vastus lateralis* muscle at the age of 33 years, that showed the presence of prominent fiber size diameter variation with multiple nuclear internalizations (Fig. 3A), mildly increased endomysial tissue (Fig. 3A, B), a homogeneous intermyofibrillar network (Fig. 3C), and fiber type 1 predominance on ATPase 9.4 reaction (Fig. 3D). Myosin Heavy Chain (MyHC) and laminin immunoflourescence confirmed the presence of global hypotrophy (Fig. 3E, F) and type 1 fibers predominance (Fig. 3F, G). PAX7/KI67 immunohistochemistry showed an increase in the number total of satellite cells, and a slight increase in their proliferation, compared to a control (Fig. 3H–J).

#### RNA and protein sudies

**Fig. 4:**
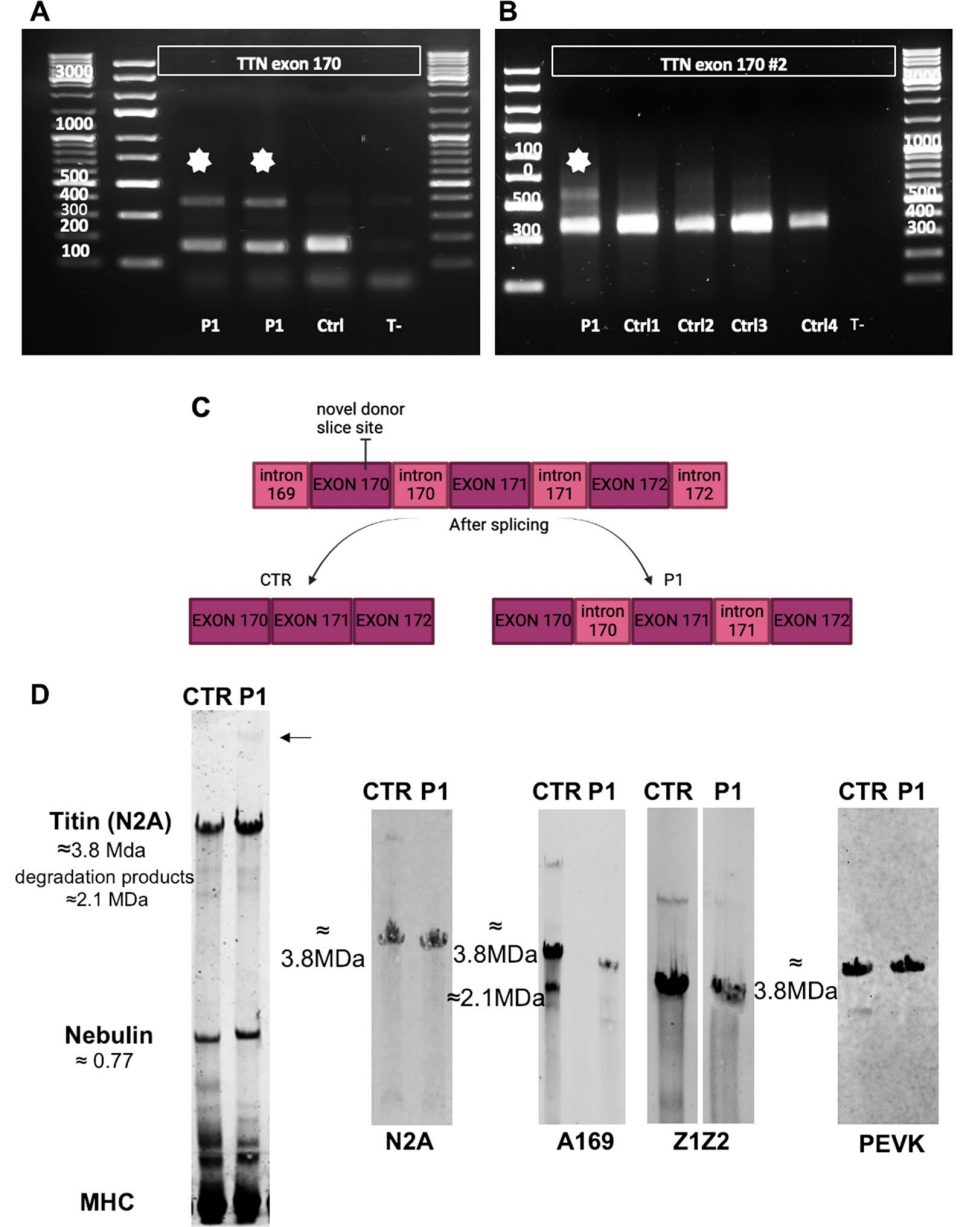
RNA and protein studies. **A**, **B** RT-PCR agarose gel electrophoresis using two different pairs primers showing a detectable band above the expected one, indicated by a white star. **C** Schematic representation of the retained introns in P1 versus CTR. **D** Muscle homogenate gel electrophoresis of P1 and a control. The molecular masses of the protein are shown on the left. The Titin degradation products, nebulin and myosin are also shown. MHC was used as a loading control. Western blot analysis of patient muscle samples using different antibodies detecting different titin domains. Anti-titin antibodies: N2A isoform (Mouse N2A T5650 USbiological), A-band (Rabbit A169 #11-96 Myomedix), Z-disk (Rabbit Z1Z2 TTN-1 Miomedix), PEVK region (Mouse PEVK 9D10 DSHB). Titin degradation products and their predicted molecular weight are indicated on the left. The data are presented as mean ± SEM

RNA studies revealed the presence of a 377BP band in titin compared to that of the normal 157BP band (Fig. 4A, indicated by stars), corresponding to the retention of intron 170 and 171 following the creation of a novel donor splice site. Using a different pair of primers (Fig. 4B, indicated by stars) spanning from exon 170 to exon 174, we confirmed the retention of the introns as schematically represented in Fig. 4C. The retention of intron 170 and 171 was further confirmed by Sanger sequencing. Immunofluorescence studies on muscle sections from P1 and a control using the of PEKV 9D10 antibody to stain against a portion of titin that is downstream of the mutated exon 170 showed a normal titin content and distribution (data not shown).

Blue Coomassie staining and PAGE electrophoresis disclosed the presence of a band of higher molecular weight above the band corresponding to the N2A isoform of titin, which is the main skeletal muscle isoform (Fig. 4D, indicated by an arrow) in P1. Western blotting using three different anitbodies against titin showed an amount of titin protein comparable to the control (Fig. 4D). These data suggest that the mutated ‘intron-retaining’ protein is present at very low levels.

#### Single fiber muscle force measurement

**Fig. 5:**
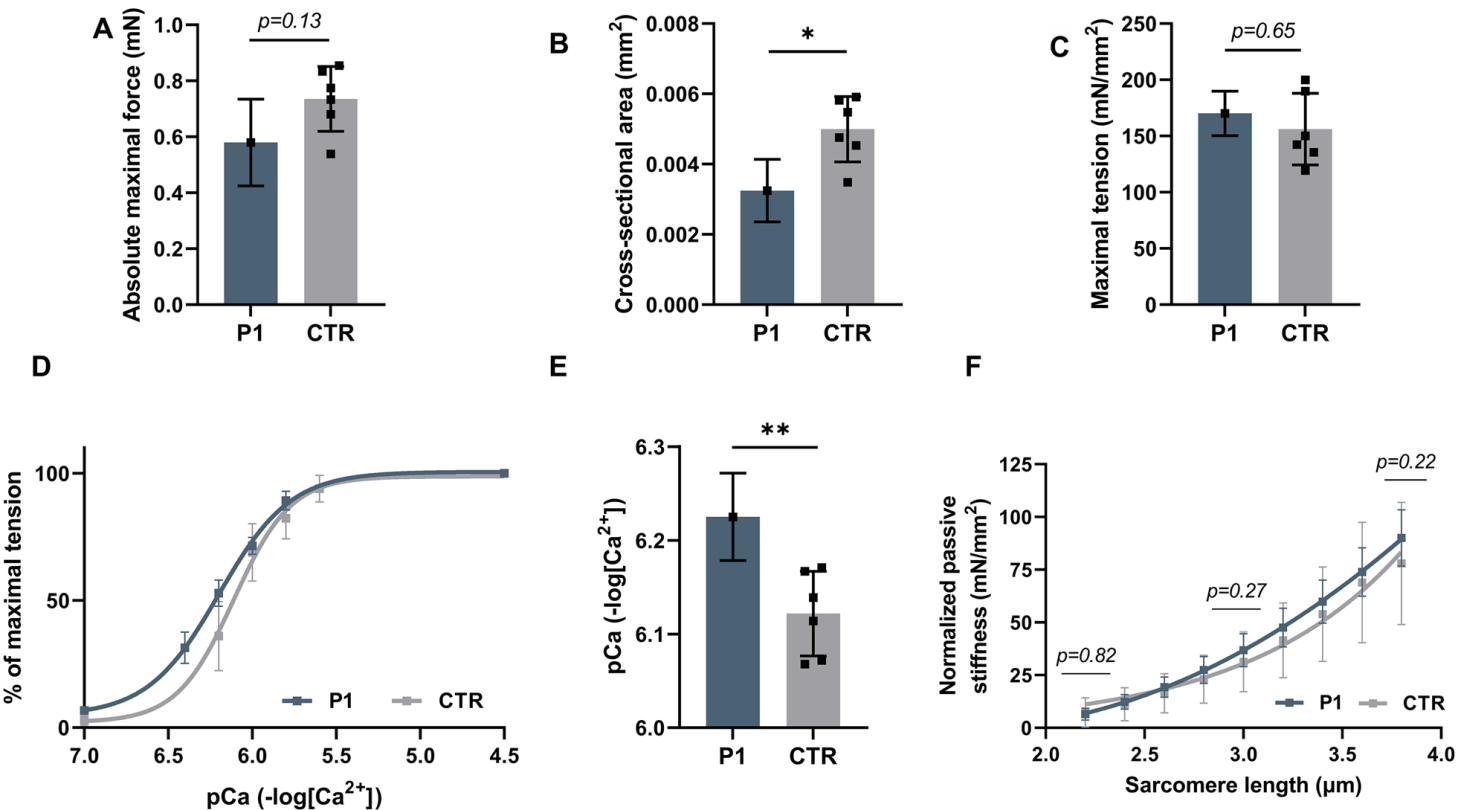
Contractile measurements of single muscle fiber from P1 (33 years old) and controls. **A** A trend towards decreased absolute force in type 1 muscle fibers of P1 (1 biopsy, *n* = 12 fibers) compared to controls (6 biopsies, *n* = 7–14 fibers/biopsy). **B** Significant decrease in CSA in type 1 muscle fibers of P1 (1 biopsy, *n* = 12 fibers) compared to controls (6 biopsies, *n* = 7–14 fibers/biopsy). **C** No significant difference in maximal normalized force in type 1 muscle fibers between P1 (1 biopsy, *n* = 12 fibers) and controls (6 biopsies, *n* = 7–14 fibers/biopsy). **D** Force-pCa curve of P1 (1 biopsy, *n* = 12 fibers) shows a leftward shift compared to controls (6 biopsies, *n* = 7–14 fibers/biopsy). **E** Significant increase in pCa50 in type 1 muscle fibers of P1 (1 biopsy, *n* = 12 fibers) compared control (6 biopsies, *n* = 7–14 fibers/biopsy). **F** No significant difference in passive stiffness of type 1 muscle between P1 (1 biopsy, *n* = 9 fibers) and controls (3 biopsies, 2–9 fibers/biopsy) at 3 different sarcomere lengths. The data are presented as mea*n* ± SD. Statistical analysis performed by linear mixed model and Wald chisquare test, *p < 0.05, **p < 0.01

Muscle fibers of P1 showed a trend towards a decreased absolute maximal force (mean difference = 0.15 mN, *p* = 0.13, Fig. 5A). To investigate whether muscle weakness experienced by the patient is sarcomere-based, the absolute maximal force was normalized to CSA (maximal tension). CSA was significantly decreased in P1 compared to controls (mean difference = 0.0017mm^2^, *p* = 0.048, Fig. 5B). No significant decrease in maximal tension was observed in P1 (Fig. 5C). These results suggest that the muscle weakness experienced by the patient is not sarcomere-based and might be caused by hypotrophy of muscle fibers. Additionally, calcium sensitivity of force generation and passive stiffness were measured in order to investigate their contribution to the contractural phenotype. First, single muscle fibers were exposed to incremental calcium solutions to determine the calcium sensitivity of force generation. The force-pCa curve of P1 shows a leftward shift, indicating an increased calcium sensitivity of force generation compared to controls (Fig. 5D). In line with these findings, there was a significant increase in the pCa_50_ value, representing an increased calcium sensitivity of force generation (mean difference = 0.10 pCa, *p* = 0.008) (Fig. 5E). No significance difference in passive stiffness was observed between P1 and controls (Fig. 5F).

## Discussion

Here we describe a 36 year-old patient with a congenital myopathy with distal arthrogryposis evolving toward a more diffuse contractile phenotype with rigid spine and moderate non-progressive muscular weakness, harboring the novel pathogenic c.36400A > T mutation in the *titin* meta-transcript-only exon 170. From a clinical stand-point similar phenotypes, but with higher degree of severity, have been previously reported in congenital titinopathies [13] and, more specifically, in children with meta-transcript-only exons titin mutations [10, 11]. To date, our patient is the longest surviving among those previously reported in the literature with a metatrasncript-only exon *titin* mutation.

Muscle MRI of the lower limbs showed the presence patchy fibro-fatty substition of specific muscles as the *semitendinosus*, in keeping with the pattern of muscle involvement that has been previously reported by us [14] and others in patients with congenital titinopathies [7–9, 13, 15]. P1 did not show overt respiratory nor cardiac involvement. This meta transcript exons have never been described to be included in the cardiac isoforms. (https://www.cardiodb.org/titin/titin_transcripts.php), and the only other patient carrying mutations in these meta-transcript exons reported to date do not present cardiac involvement [8]. However, we recommended an annual cardiac workup as a cardiomyopathy can appear in adult age in patients with skeletal muscle titinopathies [13]. Muscle morphological studies showed the presence of lesions typical of congenital titinopathies consisting of multiple nuclear internalisations, type 1 fibers predominance and micores [7, 13, 16]. Of note the minicores lesions were not observed in the *vastus lateralis* muscle biopsy at 33 years of age. This could be due to presence to a functional adaptation of muscle with the age in keeping with the gradual clinical progression after birth, and to compensation of muscle titin isoforms [7, 8, 13, 16]. The number and the activation of satellite cells was in line with the control, suggesting a conserved regenerative capacity, possibly explaining the relatively moderate muscular weakness.

The P1 *titin* mutation resides in the exon 170 belonging to exons that are described, to date, as meta-transcript-only [6], and they code for the PEVK region (Fig. 1). This region is known to be the most variable titin domain where the highest number of splicing events occur [4]. RNA studies showed the retention of two intron 170 and 171 (Fig. 4A, B), and low level of mutated transcripts. Of interest, we could show for the first time the presence of exons 170 to 174 retention in the transcript of P1 and four different histologically normal muscles used as controls (Fig. 4A, B). In previous studies and in the major database, it has been showed that exon 170 was not included in the N2A isoform [5- https://www.cardiodb.org/titin/titin_exon.php?id=171]. More recent studies showed that exon 170 has an inclusion rate of 4% in skeletal muscle [6]. This suggest that these exons might be frequently included in one of the titin adult isoforms, in contrast to what has been previously reported [4, 6, 15]. Our results underline the need to further study the splicing events occurring in these exons.

Considering that titin undergoes alternative splicing events [4] and considering that splicing variants may result in a near full-length protein [7], we demonstrated that P1 muscle has a sufficient amount of *bona fide* functional titin (Fig. 4D), thus guaranteeing the functionality of skeletal muscle, and in keeping with the non progressive muscular weakness of our patient.

Contractile measurements were performed to determine whether this novel *TTN* variant leads to sarcomere dysfunction. The decrease in CSA and absolute maximal force, but not in maximal tension, suggests that the muscle weakness experienced by the patient is not sarcomere-based. This was supported by histological findings where muscle fibers of P1 show a slight accumulation of fibrotic tissue compared to controls (Fig. 3B). Therefore, the accumulation of fibrotic tissue and the hypotrophy of muscle fibers might be an explanation for the muscle weakness experienced by P1. The increased calcium sensitivity of force generation could be a contributing factor to his contractural phenotype. A previous study showed that a heterozygous mutation in the *TPM2* gene resulted in a hypercontractile phenotype that is likely caused by increased calcium sensitivity of force generation [17]. Similar results are reported in patients with *Tpm3.12* deletions and fast *TnI* mutations [18, 19]. Increased calcium sensitivity of force generation can result in excessively sensitized cross-bridge cycling, leading to increased basal muscular tone [17].

Furthermore, no difference in passive stiffness was observed between P1 and controls. This might be explained by sufficient amounts of functional titin found in the muscle fibers of P1. The contractile measurements indicate that muscle weakness is most likely not sarcomere based. Although the novel *TTN* variant does not affect the passive properties of the sarcomere, it does affect thin filament regulation, explaining the muscle weakness experienced by the patient, whereas the increased calcium sensitivity of force generation might contribute to the rigid spine and contractual phenotype.

## Conclusions

With our study we enlarge the genetic spectrum of congenital titionpathies and we provide evidence for the causative and pathogenic role of metatranscript-only variants in a patient with congenital titinopathy, rigid spine, and non-progressive muscular weakness. The *TTN metatranscript*-only exons play a crucial role during development, but to date the splicing control mechanism of these exons is poorly understood. From our evidence, the causative effect of the disease may be post-natal notwithstanding the metatranscript-only exon. Lastly, our study highlight the importance of muscle biopsy to perform functional validation studies of unclear genetic variants in large size genes.

## Supplementary Information

**Additional file 1: Table S1.** NGS panels myopathies.

## Data Availability

Data are available upon request from the corresponding author.

## Abbreviations

TTN: Titin
P1: Patient 1
BMI: Body mass index
MRC: Medical Research Council Scale
CK: Creatine Kinase
MRI: Magnetic resonance imaging
EKG: Elecrocardiogram
ACMG: American College of Medical Genetics and Genomics
SCs: Satellite cells
BP: Base pairs
CSA: Cross sectional area
TPM2: Tropomyosin 2
TPM3.12: Tropomyosin 3
TN1: Troponin 1
TNXB: Tenascin X
VUS: Variant of uncertain significance
