# Supplementary material for: Clinical and functional characterization of a long survivor congenital titinopathy patient with a novel metatranscript-only titin variant
Source: Acta Neuropathol Commun. 2023 Mar 21;11:48. doi: 10.1186/s40478-023-01539-4 (PMC10031982; doi:10.1186/s40478-023-01539-4)
Supplement: Supplementary file 1 — Additional file 1: Table S1. NGS panels myopathies. [file 40478_2023_1539_MOESM1_ESM.docx]

| **NGS Panels myopathies** |
| --- |
| **RETRACTILE MYOPATHIES** |
| *ADAMTS2* (NM_014244.4, except exon 1) |
| *BAG3* (NM_004281.3) |
| *COL1A1* (NM_000088.3) |
| *COL1A2* (NM_000089.3) |
| *COL3A1* (NM_000090.3) |
| *COL5A1* (NM_001278074.1) |
| *COL5A2* (NM_000393.3) |
| *COL5A3* (NM_015719.3) |
| *COL6A1* (NM_001848.2) |
| *COL6A2* (NM_001849.3) |
| *COL6A3* (NM_004369.3) |
| *COL6A6* (NM_001102608.1) |
| *COL12A1* (NM_0004370.5) |
| *EMD* (NM_000117.2) |
| *FBLN5* (NM_006329.3) |
| *FHL1* (NM_001159702.2) |
| *FKBP14* (NM_017946.3) |
| *FKRP* (NM_24301.4) |
| *GAA* (NM_000152.3) |
| *HSPG2* (NM_001291860.1, NM_005529.5, except exon 1) |
| *ITGA7* (NM_001144996.1) |
| *LAMA2* (NM_000426.3) |
| *LMNA* (NM_170707.2) |
| *PLOD1* (NM_000302.3) |
| *RYR1* (NM_000540.2, except exon 91 partially covered) |
| *SEPN1* (NM_020451.2, except exon 1) |
| *STIM1* (NM_001277961.1) |
| *TNXB* (NM_019105.6) |
| *TRIM32* (NM_012210.3) |
| *TTN* (NM_001267550.1) |

Table 1S: NGS panels myopathies
